# Photophysical studies on curcumin-sophorolipid nanostructures: applications in quorum quenching and imaging

**DOI:** 10.1098/rsos.170865

**Published:** 2018-02-14

**Authors:** Sahana Vasudevan, Asmita A. Prabhune

**Affiliations:** Division of Biochemical Sciences, CSIR-National Chemical Laboratory, Dr. Homi-Bhabha Road, Pune 411008, India

**Keywords:** curcumin, sophorolipid, photophysical, quorum quenching

## Abstract

Sophorolipid biosurfactants are biodegradable, less toxic and FDA approved. The purified acidic form of sophorolipid is stimuli-responsive with self-assembling properties and used for solubilizing hydrophobic drugs. This study encapsulated curcumin (CU) with acidic sophorolipid (ASL) micelles and analysed using photophysical studies like UV-visible spectroscopy, photoluminescence (PL) spectroscopy and time-correlated single photon counting (TCSPC). TEM images have revealed ellipsoid micelles of approximately 100 nm size and were confirmed by dynamic light scattering. The bacterial fluorescence uptake studies showed the uptake of formed CUASL nanostructures into both Gram-positive and Gram-negative bacteria. They also showed quorum quenching activity against *Pseudomonas aeruginosa*. The results have demonstrated this system has potential theranostic applications.

## 1. Introduction

Biosurfactants, such as microbial glycolipids, are biodegradable, less toxic and can potentially be used to replace the conventional surfactant usage. They possess properties like anti-microbial [1], anti-biofilm [2] and anti-cancerous activity [3]. Additionally, self-assembling properties with the ability to mimic the cell membrane [4] allow them to be used as drug delivery vehicles.

Sophorolipids (SL) are a class of biosurfactants, produced by non-pathogenic yeast (predominantly *Starmerella bombicola*). Among the available biosurfactants, sophorolipids are an attractive choice because of their high yield (about 300–400 g l^−1^) and can be produced from ‘green’ resources like oleic acid, rapeseed oil, linoleic acid, cetyl alcohol, etc. They have a wide range of applications such as household detergents, heavy metal removal, emulsifying agent in food process industry, cosmetic industry and are being extensively explored in the drug delivery applications [5]. The crude mixture of the sophorolipid has predominantly two forms—namely, lactonic and acidic. The ratio of these two forms is dependent on the culture conditions and fermentation time [6]. In applications such as nanoparticles synthesis [7], the accelerated gelation of the silk fibroin, the combinatorial roles of lactonic and acidic forms are well established [8,9].

Acidic sophorolipid (ASL), a bola amphiphile, confers a surfactant property to the crude sophorolipid. The two hydrophilic ends of a hydrophobic skeleton of oleic acid contain a sophorose and a carboxylic group. This makes ASL an excellent drug delivery vehicle similar to other bola amphiphiles [10]. ASL is a simple, water-soluble, mono-component, and stimuli-responsive molecule. Recently, interest in understanding the self-assembling properties of ASL has increased and has been studied in detail using techniques like nuclear magnetic resonance (NMR), small-angle neutron scattering (SANS) and molecular dynamics (MD) simulations. The pH and the concentration play a critical role in the morphology of the ASL micelles. At a pH < 5, the ASL micelles evolve from spherical to ellipsoidal as the concentration increases from 1 to 5 w/v% [11]. Empirical evidence has shown the ASL micellar structure to have three domains: (i) an aliphatic core, (ii) an outer hydrophilic shell that contains sophorose and COOH groups, (iii) palisade layer, which is a combination of sophorose, COOH, and an aliphatic region along with water [12]. The structural understanding has paved the way in modulating ASL for drug delivery applications specifically for encapsulating hydrophobic drug molecules such as curcumin [13,14].

Curcumin, a perennial rhizome, is an active compound obtained from *Curcuma longa* (turmeric). Curcumin is biological and pharmacological active. It acts as an anti-oxidant, anti-inflammatory [15], anti-microbial [16], neuroprotective [17], anti-malarial [18], anti-metastatic [19], anti-cancer [20], and anti-angiogenic component [21,22]. Despite these activities, the poor aqueous solubility, low bioavailability, enzymatic degradation and degradability at higher pH are the factors limiting its complete use as a polypharmacological agent. Hence, there is extensive research being carried out for making this non-toxic natural hydrophobic molecule, water soluble and bioavailable [23].

Solubilization of curcumin (CU), hydrophobic small drug molecule using acidic sophorolipid and the results on its improved anti-cancerous activity was already established [13]. The bioavailability of curcumin increased 150 times in Wistar rats in the presence of crude form of sophorolipid [14].

The above studies reveal that solubilization with sophorolipid led to the fluorescence of curcumin enhanced as a consequence of increased solubility. This finding motivated us to understand the mechanism of interaction between ASL and curcumin through photophysical analysis. The photophysical properties of curcumin are extensively studied in different solvents and systems like micelles [24–27], polymeric nanoparticles [28], cyclodextrin [29–31], bovine serum albumin [32,33], liposomes [34], microcapsules [35], nanocapsules [36] and polymeric systems [37]. It is well known that the photophysical properties of this chromophore are linked with the solvent environment and proton donating ability [38]. As curcumin is water-insoluble and aggregates, it shows an entirely different absorption and fluorescence peak as compared to the solubilized form [14]. The interaction of curcumin with various carrier systems can be very well understood with spectroscopic analysis. Hence photophysical studies were employed to analyse the stability and solubility of CUASL (Curcumin in ASL micellar environment).

The enhanced stable fluorescence of CUASL can be used as bioimaging tool for the diagnostic purpose. Curcumin and its analogues have been established as a fluorescent biomarker for confocal imaging [39] by uptake studies inside mammalian cells [40–44]. Curcumin is not yet reported as a biomarker for bacterial cells. Thus, the current study was carried out using sophorolipid (ASL) encapsulated curcumin as fluorescence tagging system in bacterial cells. This system showed easy uptake by *Escherichia coli* and *Staphylococcus aureus* and showed bright fluorescence in confocal microscopy.

It was observed from the confocal micrographs that the bacterial cells (both *E. coli* and *S. aureus*), were not damaged and that they remained intact after treatment with CUASL. The anti-bacterial action of curcumin damages and ruptures the cell membrane of the bacterial cells [45]. But in our case, the cells were not ruptured, which indicated that ASL facilitated the entry of curcumin for targeted action. Both curcumin and sophorolipid have anti-biofilm activity as well as anti-quorum sensing inhibition. Hence this study extended to evaluate the quorum quenching (QQ) ability of CUASL.

With an alarming increase in the anti-microbial resistance, targeting quorum sensing (QS) is considered to be a promising alternative therapy. QQ can be understood attenuating bacterial communication using natural and synthetic small molecules [46]. For our current studies, *Pseudomonas aeruginosa*, a Gram-negative quorum sensing model organism, was tested. *Pseudomonas aeruginosa* operates through QS to infect immune-compromised patients leading to nosocomial infections. It communicates through two signal molecules, 3-oxo-C12-AHL and C4-AHL molecule [47]. Through quorum sensing, they have the ability to form biofilm and release exoproducts like pyocyanin and pyoverdine, rendering them resistant to most of the antibiotics [48]. Targeting QS signalling of *P. aeruginosa* is a promising alternative therapy to antibiotics. Curcumin as a QQ compound was first reported against *P. aeruginosa* PA01 in whole plant and animal models [49]. There are a few reports that established quorum quenching nature of curcumin against different Gram-negative quorum sensing pathogens [50–54]. Sophorolipids have also been shown to have anti-biofilm activity [2].

Here we report the entrapment of curcumin inside ASL micelles (CUASL) and analyse the stability using photophysical analysis in a concentration-dependent manner. The current studies reveal that at the optimum concentration of 5 w/v%, acidic sophorolipid can encapsulate curcumin. The solubility is achieved at the acidic pH, where curcumin is stable, thus reducing the degradation of curcumin. The decay kinetic profile follows triple exponential decay with an average decay time of 318.5 ps, revealing that curcumin may be present in the palisade layer of the acidic sophorolipid micelle. We have demonstrated quorum quenching activity against *P. aeruginosa* and fluorescent uptake studies for imaging bacterial cells like *E. coli* and *S. aureus*. Thus, leading a way for the development of soluble curcumin-based bioimaging system.

## 2. Material and methods

### 2.1. Purification of acidic sophorolipid

**Figure 1.**
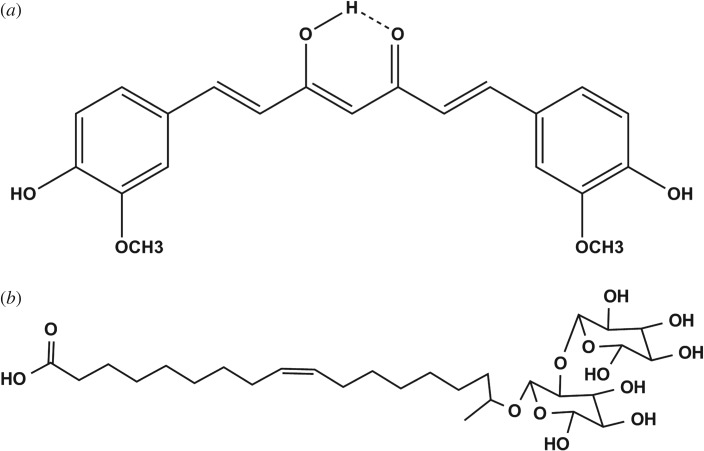
Chemical structures of (*a*) curcumin and (*b*) acidic sophorolipid.

Resting cell method was adopted to synthesize oleic acid-derived sophorolipid (OASL) by *Starmerella bombicola* ATCC 22214 [5]. The extraction and purification protocol is explained in detail elsewhere [5]. ASL was purified using alkaline hydrolysis method [9], from the crude sophorolipid which is a combination of lactonic and acidic forms of sophorolipid [55]. The purity of the obtained ASL was confirmed using H^1^ NMR-spectroscopy. The chemical structure of ASL is shown in figure 1*b*.

### 2.2. Synthesis of curcumin sophorolipid nanostructures

CU (figure 1*a*) was purchased from Sigma Aldrich (approx. 99% purity). Curcumin (1 mg ml^−1^) was probe-sonicated for 40 min using Branson Digital Sonifier 250 together with different concentrations of ASL from 1 to 5 w/v% (above CMC = 0.11 mg ml^−1^)[56].

### 2.3. Spectroscopic measurements

By keeping the concentration of curcumin constant (1 mg ml^−1^), the absorption spectra at different ASL concentrations (1–5 w/v%) were recorded by the UV-1800 Shimadzu spectrophotometer with 10 mm quartz cell. The steady-state fluorescence was measured using Shimadzu RF-6000 Spectrofluorometer. CUASL was excited at the wavelength of 420 nm to record the emission spectrum.

To check the extent of degradation of curcumin inside the acidic sophorolipid micelles, the optical density (OD) of the maximum peak was recorded at the 0th and 60th min, at different concentrations of the micelle. The degradation percentage was calculated by the formula:

2.1 $$\%\text{ degradation}=\left[\frac{{\text{OD}}_{0\;\min}-{\text{OD}}_{60\;\min}}{{\text{OD}}_{0\;\min}}\right]\times 100.$$

### 2.4. Lifetime and quantum yield measurements

Time correlated single photon counting (TCSPC) spectrometer (Horiba Jobin Yvon IBH, UK) was used to collect time-resolved fluorescence measurements. The detailed description of the instrument is explained elsewhere [57]. In the present work, the excitation source used was a 400 nm diode laser (approx. 130 ps, 1 MHz repetition rate) and an MCP-PMT (microchannel plate--photomultiplier tube) detector was used for collecting the fluorescence signal. The analysis of the lifetime was done by using IBH DAS6 analysis software. For a good fit, the *χ*^2^ value was close to unity.

The fluorescence quantum yield was calculated with quinine sulphate in 0.1 M H_2_SO_4_ (excitation wavelength= 350 nm) as a reference. Quantum yield was calculated by:

2.2 $$\Phi =(\Phi_{R})\times \left(\frac{I}{I_{R}}\right)\times \left(\frac{A_{R}}{A}\right)\times \left(\frac{\eta^{2}}{\eta_{R}^{2}}\right),$$

where R refers to the reference. *Φ* and *Φ*_R_ are the quantum yields, *I* and *I*_R_ are the fluorescence intensities, *A* and *A*_R_ are the absorbances and *η* and *η*_R_ are the refractive index.

The average fluorescence lifetime is the sum of the product of the amplitudes and the respective lifetimes and was calculated using the equation (table 1):

2.3 $$\tau_{\mathrm{av}}=a_{1}\tau_{1}+a_{2}\tau_{2}+a_{3}\tau_{3}.$$

The rate constants for the radiative decay (*k*_r_) and non-radiative decay (*k*_nr_) were calculated.

2.4 $$k_{r}=\frac{\Phi_{f}}{\tau_{\mathrm{av}}}$$

and

2.5 $$k_{\mathrm{nr}}=\left(\frac{1}{\tau_{\mathrm{av}}}\right)-k_{r}.$$

**Table 1.** Lifetime measurement parameters of CUASL (5 w/v% ASL concentration).

CUASL (w/v%)	*τ*_1_ (ps)	*a*_1_	*τ*_2_ (ps)	*a*_2_	*τ*_3_ (ps)	*a*_3_	*τ*_av_ (ps)	*χ*^2^
0	18.5	0.66	106.7	0.33	523	0.01	52.651	0.98
5	89	0.54	406	0.32	1003	0.14	318.4	1.08

### 2.5. Size distribution and stability

#### 2.5.1. Transmission electron microscopy

The transmission electron microscopy (TEM) images of the CUASL solution were observed using FEI Technai G2 120 kV instrument. The samples were diluted 100 times and were drop-cast in a carbon-coated copper grid (mesh size 200) from Icon Analyticals Pvt. Ltd, India and air-dried overnight.

#### 2.5.2. Zeta potential and dynamic light scattering

The zeta potential and particle size measurements were done on the Brookhaven Instrument model 90 Plus Particle Size Analyzer and potential analyser. The zeta potential and average particle size of the CUASL nanostructures were determined with 100 times dilution.

### 2.6. Confocal microscopy

Laser scanning confocal microscopy from Zeiss was used for observing the stained cells. The microorganisms taken for this study were *S. aureus* ATCC 29737 (Gram-positive), *E. coli* NCIM 5129. The overnight culture of the above microorganisms was set to OD 0.1. The cells were exposed to the fluorescent CUASL (5 w/v%) for not more than 2 h. The cells were washed to remove the unbound nanostructures and mounted on the coverslip, and glycerol was used as the mounting medium. The coverslip was sealed to prevent drying by evaporation. The slides were prepared freshly and visualized within 5 h. The excitation wavelength was 488 nm and emission was recorded at the wavelength range of 500–600 nm.

The images were processed using the Zen 2010 and ImageJ software. The fluorescence intensity of CUASL-treated cells, CTCF (corrected total cell fluorescence) was calculated using the following formula:

2.6 CTCF=integrated density−(area of selected cell×mean fluorescence of background readings).

### 2.7. Quorum quenching assay

#### 2.7.1. Bioluminescent reporter assay

Bioluminescence expression was quantified using a Tecan luminometer (Infinite M200, Männerdorf, Switzerland). An overnight culture of *E. coli* biosensor cells, pSB1142 (A kind gift from Prof. Williams, University of Nottingham, UK) was set to an OD600 nm of 0.2. Then, 200 µl of *E. coli* biosensor cells and CUASL (5 w/v%) having different concentrations of curcumin from 10 to 50 µg ml^−1^ and ASL (5 w/v%) control were added into the well of 96-well microtiter plate. Expression of bioluminescence was measured in terms of relative light unit (RLU)/OD495 nm. Bioluminescence reduction in *E. coli* [pSB1142] explains the anti-QS properties of the CUASL nanostructures. CUASL (5 w/v%) and ASL (5 w/v%) at their respective concentrations were taken as control.

The quorum quenching activity of CUASL against *Pseudomonas aeruginosa* was done with the following assays. *Pseudomonas aeruginosa* NCIM 5029, used for the QQ assays, were grown in Luria Bertani media (LB) and maintained in glycerol stock at −20°C. ASL (5 w/v%) was taken as control for all the assays.

#### 2.7.2. Anti-biofilm assay

CV biofilm assay was done in 96-well microtiter plate (MTP), as mentioned previously [58]. CUASL (5 w/v%) having different concentrations of curcumin from 10 to 50 µg ml^−1^ were tested against biofilm formation at the 8th hr. The time point of the 8th hr was chosen because it is shown that the biofilm formation initiates at the 8th hr [59]. 200 µl of an overnight culture of *P. aeruginosa* was added to each well, set to OD 0.1 at 600 nm. The planktonic cells were removed and washed with distilled water twice and 200 µl of 0.1% crystal violet solution was added, and incubated for 15 min at room temperature. The excess crystal violet solution was discarded and 200 µl of 30% acetic acid solution was added to each of the wells. The reading was recorded at the absorbance of 580 nm.

#### 2.7.3. Auto-aggregation assay

To understand the anti-adherence activity of CUASL, an overnight culture of *P. aeruginosa* was set to 0.1 OD at 660 nm. For this assay, the readings were recorded at the 8th hr. The OD at 660 nm at the 0th min was recorded and incubated at room temperature for 60 min. The OD at the 60th min was recorded at 660 nm. The auto-aggregation index was calculated as:

2.7 $$\text{auto-aggregation index}=\left(\frac{{\text{OD}}_{0\;\min}-{\text{OD}}_{60\;\min}}{{\text{OD}}_{0\;\min}}\right).$$

#### 2.7.4. Pyoverdine

**Figure 2.**
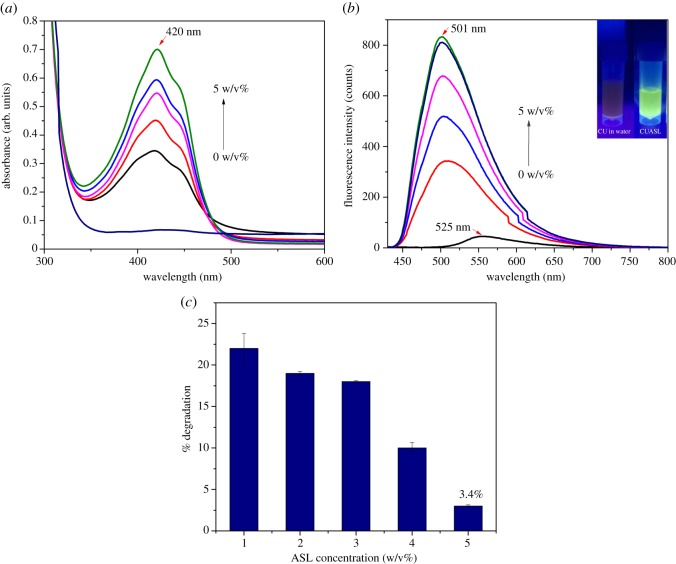
(*a*) Absorbance spectrum of CUASL at different ASL concentrations. As the concentration of ASL increases there is an increase in the intensity and peak shift to approximately 420 nm. (*b*) Emission spectrum of CUASL at different concentrations of ASL. A 24 nm peak shift can be observed with the increasing concentrations of ASL. Inset: green fluorescence of CUASL shown under UV transilluminator. (*c*) Degradation studies of curcumin at different ASL concentrations. At 5 w/v% of ASL, curcumin's degradation rate was reduced.

The fluorescent siderophore, pyoverdine, is a quorum sensing mediated phenotype produced by *P. aeruginosa*. The inhibition of pyoverdine production was calculated by measuring the fluorescence. In 96-well MTP, 200 µL of *P. aeruginosa* (OD set to 0.1 at 600 nm) treated with CUASL at different concentrations (10–50 µg ml^−1^) was added. The excitation wavelength of CUASL falls in the range of 470–570 nm (figure 2*b*), CUASL did not contribute to the measured pyoverdine fluorescence. The cells were excited at 405 nm and emission was recorded at 465 nm [60].

#### 2.7.5. Pyocyanin

Pyocyanin is another exoproduct of *P. aeruginosa* synthesized by quorum sensing signalling. The overnight culture was set to 0.2 OD at 600 nm and CUASL was added at different concentrations (10–50 µg ml^−1^). The extraction of pyocyanin was done at the 24th hr by the following procedure. To the 5 ml of the cell-free supernatant, 3 ml chloroform and 1 ml 0.2 M HCl were added and centrifuged for 20 min at 8000 r.p.m. (28°C). The HCl layer was carefully separated and measured at OD 520 nm, keeping 0.2 M HCl as blank [61].

## 3. Results

### 3.1. Spectroscopic analysis

The spectrophotometric effect of ASL on curcumin was studied systematically at different concentrations from 1 to 5 w/v% (figure 2*a*). Structurally curcumin is a diferuloyl methane (figure 1) and exists as keto-enol isomer. In solutions, the enol form dominates the keto form [34]. A striking change in the absorption spectrum was observed in CUASL after the addition of ASL with increasing concentrations. The conjugated diferuloyl structure corresponds to the enol form, which is visualized at approximately 420 nm peak. The maximum absorption peak at approximately 420 nm indicates that it is due to the low-energy π-π* transition and curcumin is in its enolic form [62]. Curcumin is insoluble in water and hence absorption peak cannot be seen clearly, a similar trend for the curcumin in water was seen in our previous study [14]. A small shoulder at 440 nm can be observed in the presence of acidic sophorolipid. The peak shift and the 440 nm shoulder peak, as can be seen also in similar micellar systems, can be attributed to the shifting of curcumin from bulk to the micellar environment [24–27]. As previous literature suggests [24], this points us towards the location of curcumin in the palisade layer of the ASL micelles.

Steady-state fluorescence studies showed that the intensity of the curcumin fluorescence increases with the increase in the concentration of ASL (figure 2*b*). There was a blue shift (Δ*λ*_fl _= 24 nm) in the fluorescence maximum (*λ*_fl_) of CUASL (*λ*_fl _= 501 nm) when compared with curcumin in water (*λ*_fl _= 525 nm). This 24 nm blue shift in the emission spectrum indicated that curcumin is moving from the bulk phase into the micellar environment of ASL micelles. This blue shift can also be interpreted as hydrophobic interactions between curcumin and ASL micelles, similar to other micellar environments, especially TX-100 (*λ*_fl _= 501 nm) [24].

When encapsulated with ASL micelles (pH ∼4.5), curcumin was completely soluble, as opposed to the usual solubility of curcumin which is only possible at alkaline pH. A similar result was obtained when curcumin was solubilized in rhamnolipid micelles [63]. Rhamnolipids are glycolipid biosurfactants and are structurally similar to sophorolipids. This recent study showed that the interaction between the rhamnolipid and curcumin is pH dependent, and at acidic pH curcumin's stability was maintained. It has been understood that curcumin is stable at a pH range 1–6, because of the undissociated form of the hydroxyl groups [64]. About 80% of curcumin degrades in the aqueous buffer (pH 7.4) in a period of 60 min into vanillin, ferulic acid and feruloyl methane [65]. This degradation is attributed to the decrease in the absorption value. Thus, the degradation studies were conducted by recording the OD at the 0th and 60th min for different concentrations of ASL, to show that the acidic sophorolipid micelles minimize degradation of curcumin (figure 2*c*). At the concentration of 5 w/v%, the degradation of curcumin was as low as 3.4% and at the minimum concentration of 1 w/v%, 22.2%. Thus the spectroscopic and degradation studies showed that at 5 w/v%, curcumin was stable and the interaction with bulk water was minimized because of its encapsulation inside the ASL micelles. In addition to encapsulation, the pH of the ASL micellar system also contributes to the stability and reduced degradation of curcumin.

The above results revealed that the stability of the curcumin inside ASL micelles was concentration dependent and that 5 w/v% ASL was more stable with enhanced fluorescence. Thus, ASL micelles act as a drug carrier providing a stable environment for curcumin.

### 3.2. Lifetime and quantum yield measurements

Quantum yield and lifetime measurements are essential characteristics of a fluorophore. While quantum yield gives the information on the number of emitted photons relative to the absorbed photons, lifetime measurements provide the interaction time of the fluorophore with its environment. TCSPC is an excellent tool to understand the excited state dynamics. It is also useful to know the strength, stability and the layer in which the drug is bound to the micelle. The time-resolved study gives an idea of the drug location in the micelle and also details regarding the surrounding environment of the drug.

The quantum yield of curcumin-sophorolipid nanostructures at 5 w/v% was calculated by taking quinine sulphate in 0.1 M H_2_SO_4_ as the standard [38] and was found to be 0.014. Because of the effect of non-radiative decay, there was no marked difference in the quantum yield at different ASL concentrations. This observation was complemented by the lifetime measurements, as not much lifetime difference was witnessed at different ASL concentrations.

**Figure 3.**
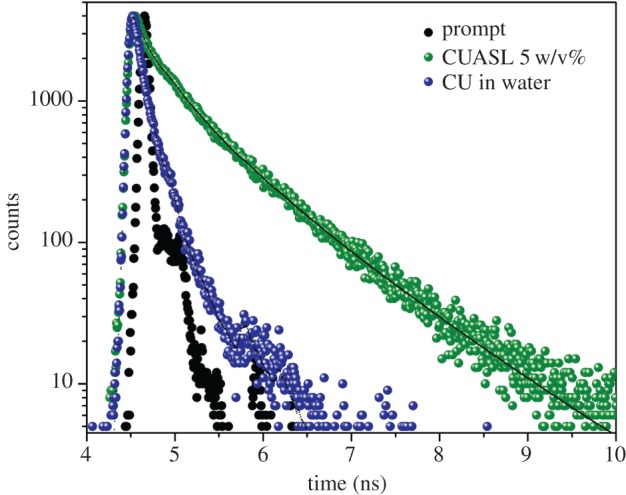
Lifetime measurement of CUASL (5 w/v%) and CU in water. There is an increase in lifetime with triple exponential decay in the presence of 5 w/v% ASL.

The decay profile (figure 3) of curcumin entrapped in 5 w/v% ASL micelles and water was recorded at 501 nm using the 400 nm diode laser source. As it is evident from the decay profile that curcumin in water is overlapped with the prompt, hence the decay is negligible. The decay for 5 w/v% was multi-exponential and was fit using triple exponentials. The average lifetime of CUASL was 318.5 ps with the fastest decay component at 89 ps, the slower component at 406 ps and longer-lived component at 1003 ps. The multiexponential nature of the decay indicates a complex nature of the interaction between curcumin and ASL micelles. This corroborates well with the absorption studies and confirmed that curcumin was entrapped in the palisade layer of the acidic sophorolipid micelles which is a mixed combination of sophorose, COOH, and aliphatic region along with water.

It is well known that the major photophysical event of curcumin is excited state intramolecular proton transfer (ESIPT). In the presence of non-polar solvents, the ESIPT process increases leading to a very short lifetime (few hundred femtoseconds). On the other hand, in the polar solvents like acetone, the ESIPT process decreases and thereby increases the lifetime. A similar trend was seen in the case of CUASL nanostructures, the ESIPT was minimized, as it can be reflected by the longer lifetimes (figure 3). The increase in the lifetime was due to the ESIPT process hindrance that may be due to the intermolecular hydrogen bonding or hydrophobic interaction between the oleic acid moiety of the ASL and curcumin as pointed out previously [13].

The radiative and non-radiative decay kinetics were calculated, and the photophysical parameters were tabulated (table 2). The relatively low non-radiative decay constant (*k*_nr_) and increase in the radiative decay constant (*k*_r_) reinforces the hindrance in the ESIPT process after the addition of ASL. The results obtained are comparable with the polymeric micelles [27,28]. The higher lifetime value and amplified fluorescence intensity of CUASL, as compared to CU in water, can be ascribed to the increased radiative decay value (*k*_r_).

**Table 2.** Photophysical parameters of CUASL (5 w/v% ASL concentration).

CUASL (w/v%)	*λ*_a_ (nm)	*λ*_fl_ (nm)	*φ*_f_	*τ*_av_ (ps)	*k*_r_/10^7^ (s^−1^)	*k*_nr_/10^9^ (s^−1^)
0	430	525	—	52.651	—	—
5	420	501	0.014	318.5	4.39	3.09

### 3.3. Size distribution

**Figure 4.**
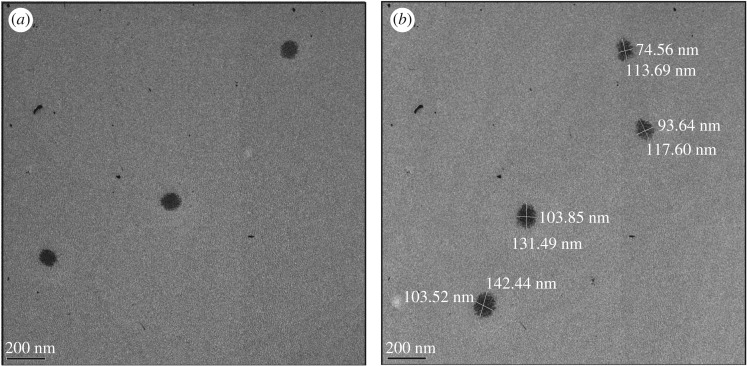
(*a*,*b*) TEM images of the formed CUASL nanostructures.

The formed nanostructures were intact as seen from the TEM images (figure 4). The formed CUASL micelles were not perfectly spherical, rather a little elongated, forming ellipsoidal structures were approximately 100 nm size.

The above size was confirmed with dynamic light scattering (electronic supplementary material, figure S1) and the size distribution of around 100 nm with the polydispersity index of 0.27. This size was calculated in the solution whereas TEM characterization was done with air-dried sample. The polydispersity index and non-uniformity of TEM images confirmed that the micelles were dynamic, giving minor changes in the overall size of the nanostructures.

The stability of CUASL nanostructures was measured using zeta potential (electronic supplementary material, figure S1). The average zeta potential was −38.41 mV which indicated that the CUASL structures were quite stable. The above size distribution and stability analysis confirmed that formed CUASL nanostructures and their stability are complementary to the photophysical analysis.

**Figure 5.**
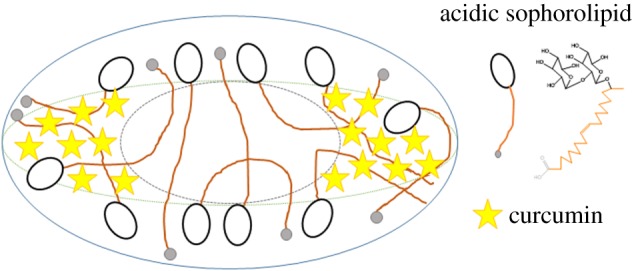
Schematic diagram representing the probable position of curcumin within the palisade layer of ASL micelles. ASL micelle structure was redrawn as given in the reference [12].

The schematic representation of the CUASL nanostructures is shown in figure 5.

### 3.4. Confocal microscopy

**Figure 6.**
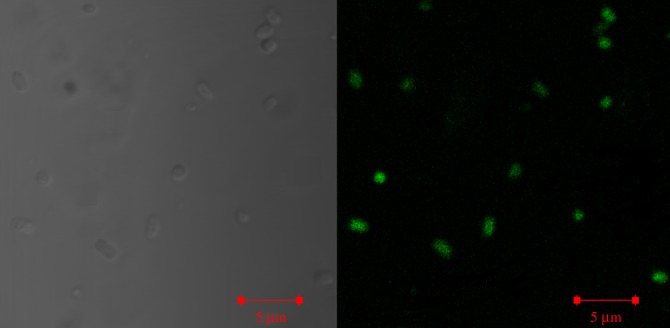
Confocal micrographs of *E. coli* treated with CUASL (5 w/v%). Left is the phase contrast image and right is the fluorescent image of the bacterial cells. The CUASL treatment has not ruptured the cells.

**Figure 7.**
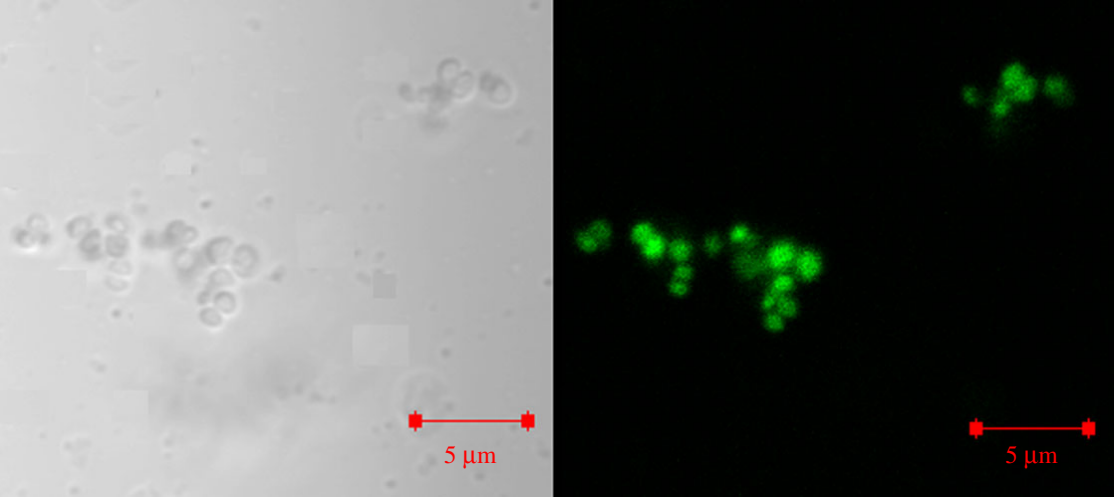
Confocal micrographs of *S. aureus* treated with CUASL (5 w/v%). Left is the phase contrast image and right is the fluorescent image of the bacterial cells. The CUASL treatment has not ruptured the cells.

The photophysical studies revealed that CUASL nanostructures have enhanced green fluorescence, which can be exploited for bioimaging applications. The confocal microscopic analysis was carried out to investigate the uptake of CUASL nanostructures by the bacterial cells. The confocal microscopic images (figures 6 and 7) showed bright green fluorescent bacterial cells. In the short time span of 2 h, the bacterial cells (both Gram-negative and Gram-positive) effectively internalized CUASL nanostructures.

CTCF was calculated for treated and untreated cells, and since untreated cells do not show fluorescence, the CTCF was near zero. And for the CUASL-treated cells, CTCF was calculated to be 48 241 intensity units for *E. coli* and 32 541 intensity units for *S. aureus*. The previous study on the synergistic action of sophorolipids and antibiotics speculated that the sophorolipids facilitate the entry of the antibiotics into the bacterial cell interior [66]. A similar mechanism can be extrapolated to CUASL nanostructures. Acidic sophorolipids have self-assembling properties and mimic the bacterial cell membrane. This helps to span through the cell membrane and thereby release curcumin inside the cytoplasm of the cell. It is interesting to note that the CUASL-treated cells, did not show any change in morphology, which visually indicates that the fluorescent CUASL nanostructures did not rupture the cells, and hence can be effectively used for bioimaging.

Curcumin was shown to be used as a fluorescent biomarker in mammalian uptake studies by using different solvents [40–44]. Though the quantum yield was low, bacterial cell uptake studies showed that CUASL can be tagged with specific markers; it can be used for selective imaging of different types of cells, exploring its use as a bioimaging tool.

Since the bacterial cells remained intact after the treatment of CUASL, it leads us to explore its biological activity.

### 3.5. Quorum quenching assays

#### 3.5.1. Bioluminescent assay

The CUASL (5 w/v%) showed a significant reduction in bioluminescence, with the maximum inhibition at the concentration of 50 µg ml^−1^. The signalling molecule used was 3-oxo-C12 HSL, which is specific to *P. aeruginosa.* The CUASL showed effective inhibition of 3-oxo-C12 HSL (electronic supplementary material, figure S2). This indicated that CUASL was bound to the signal receptor. It should be noted that ASL (5 w/v%) did not have any quorum quenching effect (data not shown). This helped us to proceed for checking quorum quenching activity of CUASL against *P. aeruginosa*.

#### 3.5.2. Anti-biofilm and anti-adherence activity

**Figure 8.**
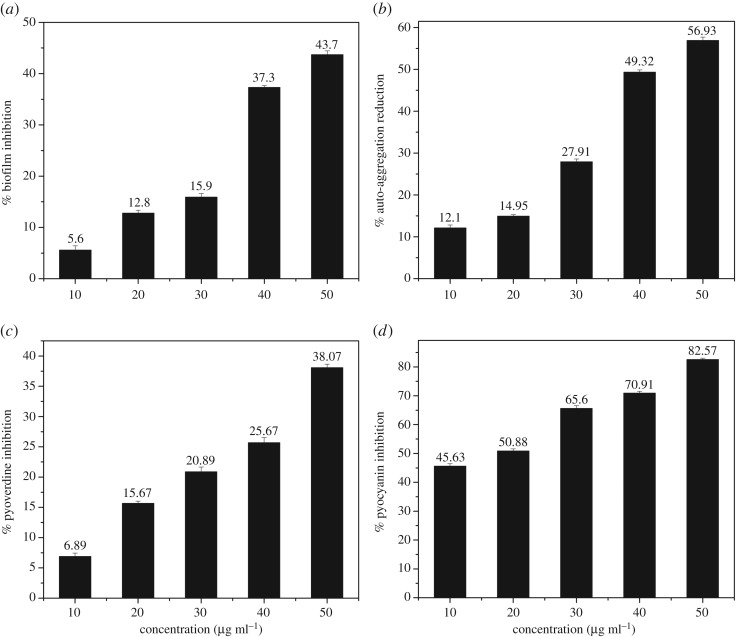
Quorum Quenching Activity of CUASL (5 w/v%) against *P. aeruginosa*: (*a*) anti-biofilm activity, (*b*) auto-aggregation reduction activity, (*c*) pyoverdine reduction, (*d*) pyocyanin reduction.

The ability to form biofilm by bacteria is considered to be the most efficient escape mechanism from antibiotics. Biofilm formation is mediated through quorum sensing pathways. The biofilm formation for *P. aeruginosa* initiates at the 8th hr. Thus, for anti-biofilm activity, CUASL should act before the commencement of biofilm formation. The maximum biofilm inhibition of CUASL was recorded at 50 µg ml^−1^ at the 8th hr. Previous reports have already proved that curcumin has anti-biofilm effects when dissolved in different solvents; ASL (5 w/v%) was taken as control and it did not have a significant anti-biofilm effect (data not shown) (figure 8*a*).

To understand the effect of adherence, auto-aggregation index was calculated at the same time point and concentration, as that of a biofilm. The auto-aggregation index matched exactly with the anti-biofilm activity. At the 8th hr and at 50 µg ml^−1^ concentration of curcumin, 58% inhibition was observed in auto-aggregation. Thereby, the bacterial cells aggregation was inhibited (figure 8*b*). Bacterial aggregation leads to the biofilm formation. Thus CUASL acts as an anti-biofilm agent by perturbing the aggregation process.

#### 3.5.3. Pyocyanin and pyoverdine assay

The exoproducts released by *P. aeruginosa* play a significant role in rendering resistance to antibiotics. The siderophore pyoverdine is an iron transporter which is linked to the biofilm formation and mediated through quorum sensing. As CUASL showed anti-biofilm activity, it is quite likely to perturb the pyoverdine synthesis as well. Hence the effect of CUASL on pyoverdine inhibition was tested. The CUASL facilitated pyoverdine inhibition at 50 µg ml^−1^ concentration in 24 h. The decrease in the auto-fluorescence mediated by pyoverdine was observed (figure 8*c*).

Pyocyanin is a phenazine found in the sputum of *P. aeruginosa* infected patients. Its production is regulated under quorum sensing signalling. It generates reactive oxygen species and thus creates oxidative stress in healthy cells causing damage. When CUASL was tested against pyocyanin production, at 50 µg ml^−1^ concentration of CUASL, pyocyanin was inhibited by almost 82% (figure 8*d*).

The virulent factors regulated by quorum sensing pathways of *P. aeruginosa* were attenuated by CUASL nanostructures, without inhibiting the growth. This suggests that CUASL nanostructures showed quorum quenching activity against *P. aeruginosa*.

## 4. Conclusion

The present study reports the use of acidic sophorolipid for solubilizing the hydrophobic drug, curcumin, by entrapping them inside micelles. The CUASL system was analysed using photophysical techniques like UV-Vis spectroscopy, fluorescence spectroscopy and lifetime measurements. In addition to making curcumin aqueous and soluble at an acidic pH, we have shown that ASL reduces the degradation of curcumin by hydrolysis by almost 95%. The enhanced green fluorescence of CUASL with the blue shift signifies the movement of curcumin into the ASL micellar environment. Lifetime and quantum yield measurements reveal that the encapsulation by ASL micelles changes the excited state dynamics and leads to the reduction in ESIPT (excited state intramolecular proton transfer). Since it was already shown in the previous literature that curcumin, as well as sophorolipid, have anti-biofilm activity, we have demonstrated that they work synergistically as a quorum quencher against *Pseudomonas aeruginosa* and finally, the property of fluorescence was extended as a biomarker agent for confocal imaging. The bright green fluorescence imparted to the bacterial cells opens up an area for the development of soluble curcumin-based bioimaging system. This is the first report of photophysical analysis of curcumin encapsulated inside biosurfactant micelles.

## Supplementary Material

Size Distribution ;Quorum Quenching Assays

## Data accessibility

The datasets, electronic supplementary material, figure S1: Zeta potential and DLS log summary of CUASL and electronic supplementary material, figure S2: Quorum quenching-Bioluminescence assay of CUASL supporting this article have been uploaded as part of the electronic supplementary material.
