# Supplementary material for: Photophysical studies on curcumin-sophorolipid nanostructures: applications in quorum quenching and imaging
Source: R Soc Open Sci. 2018 Feb 14;5(2):170865. doi: 10.1098/rsos.170865 (PMC5830715; doi:10.1098/rsos.170865)
Supplement: Size Distribution ;Quorum Quenching Assays [file rsos170865supp1.docx]

**Supporting Information**

Photophysical studies on Curcumin-Sophorolipid Nanostructures: Applications in quorum quenching and imaging

Sahana Vasudevan and Asmita A. Prabhune*

Division of Biochemical Sciences, CSIR- National Chemical Laboratory, Pune-411008, India.


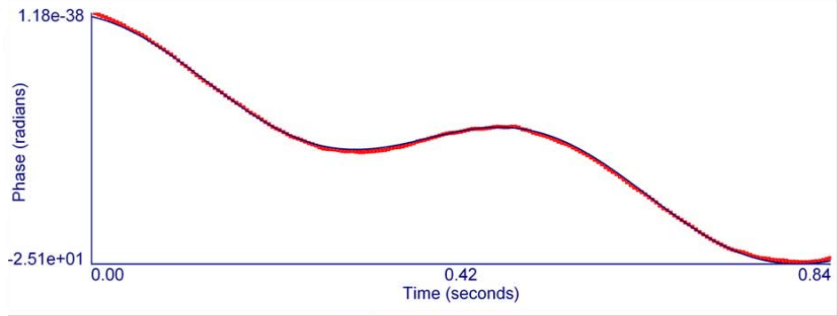

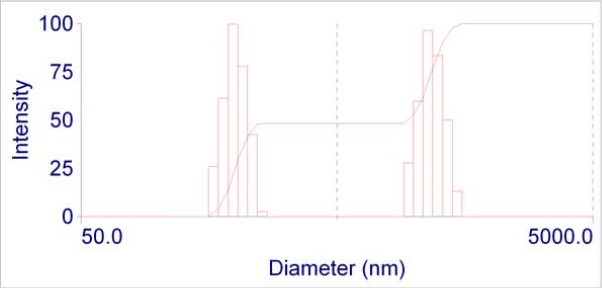


**Figure S1: Zeta Potential (-38.41mV) and DLS (100nm, polydispersity index: 0.27) of CUASL (5w/v %)**





**Figure S2: Bioluminescence reduction by CUASL (5w/v %)**
